# Supplementary figures and images for: Targeting the Sequences of Circulating Tumor DNA of Cholangiocarcinomas and Its Applications and Limitations in Clinical Practice
Source: Int J Mol Sci. 2023 Apr 19;24(8):7512. doi: 10.3390/ijms24087512 (PMC10144736; doi:10.3390/ijms24087512)

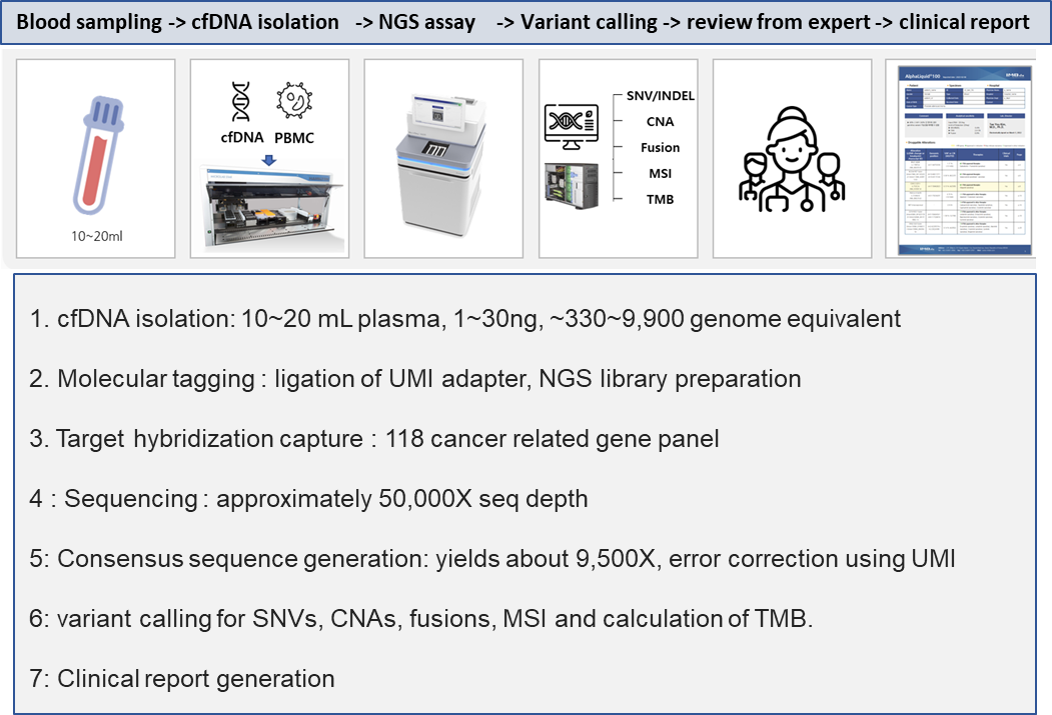

Supplement: Supplementary file 1 [file ijms-24-07512-s001.zip › Supplementary figure.tif]
